# Supplementary material for: Ultrafast piezocapacitive soft pressure sensors with over 10 kHz bandwidth via bonded microstructured interfaces
Source: Nat Commun. 2024 Apr 8;15:3048. doi: 10.1038/s41467-024-47408-z (PMC11001880; doi:10.1038/s41467-024-47408-z)
Supplement: Supplementary file 1 — Supplementary information [file 41467_2024_47408_MOESM1_ESM.pdf]

Supporting Information

Ultrafast piezocapacitive soft pressure sensors with over 10 kHz bandwidth via bonded microstructured interfaces

Yuan Zhang[1,#], Xiaomeng Zhou[2,#], Nian Zhang[3,#], Jiaqi Zhu[1], Ningning Bai[1], Xingyu Hou[1], Tao Sun[4], Gang Li[1], Lingyu Zhao[1], Yingchun Chen[5,*], Liu Wang[3,6,*], Chuan Fei Guo[1,*]

[1]Department of Materials Science and Engineering, Southern University of Science and Technology, Shenzhen 518055, China

[2]CAS Key Laboratory of Human-Machine Intelligence-Synergy Systems, Shenzhen Institutes of Advanced Technology Chinese Academy of Sciences, Shenzhen 518055, China

[3]CAS Key Laboratory of Mechanical Behavior and Design of Materials, University of Science and Technology of China, Hefei 230000, China

[4]Department of Computer Science and Engineering, Southern University of Science and Technology, Shenzhen 518055, China

[5]Science and Technology Committee, Commercial Aircraft Corporation of China Ltd., Shanghai 200126, China

[6]State Key Laboratory of Nonlinear Mechanics, Institute of Mechanics, Chinese Academy of Science, Beijing 100190, China

[#]These authors contributed equally to this work.

*To whom correspondence should be addressed. E-mail: guocf@sustech.edu.cn (C.F.G.), wangliu05@ustc.edu.cn (L.W) or chenyingchun@comac.cc (Y.C.)

The file includes:

Supplementary Figure S1. SEM images of the template made by 3D printing.
Supplementary Figure S2. Schematic diagram illustrating the preparation process of the bonded interface.
Supplementary Figure S3. Finite element simulations of both non-bonded and bonded pressure sensors under the unloading process.
Supplementary Figure S4. Schematic design diagram of the digital circuit board.
Supplementary Figure S5. Diagram of power supply module and microcontroller module of the digital circuit board.
Supplementary Figure S6. SEM images of sensor structures with biomimetic structures and 3D printed structures.
Supplementary Figure S7. Adhesion force between a PDMS cone and a PDMS thick layer during contact, pressing, and releasing.
Supplementary Figure S8. Peel forces as a function of displacement for the three samples with bonded microstructured interface by 180° peeling.
Supplementary Figure S9. Capacitive response of the sensor to 60,000 cycles of vibrations at a frequency of 12500 Hz.
Supplementary Figure S10. Capacitive response of the pressure sensor to mechanical vibrations of different amplitudes at a fixed frequency of 12500 Hz.
Supplementary Figure S11. Responses in both time and frequency domains of the sensors with and without interfacial bonding under vibrations of different frequencies.
Supplementary Figure S12. Frequency spectra of the signals under a static pressure (100 kPa) and superposed vibrations of different frequencies.
Supplementary Figure S13. Responses of our sensor to superposed vibrations.
Supplementary Figure S14. Capacitance responses of the sensor to sounds of different sound pressure levels.
Supplementary Figure S15. Experimental data and fitting results for the relative permittivity of the dielectric layer material with respect to pressure.
Supplementary Figure S16. Calculation of geometric dimensions and capacitance for bonded micro-cone structure.
Supplementary Figure S17. Geometric dimensions and capacitance calculation before and after the compressed loading process of the bonded micro-cone structure.
Supplementary Text 1. The design of digital circuit board.
Supplementary Text 2. Finite element simulation.

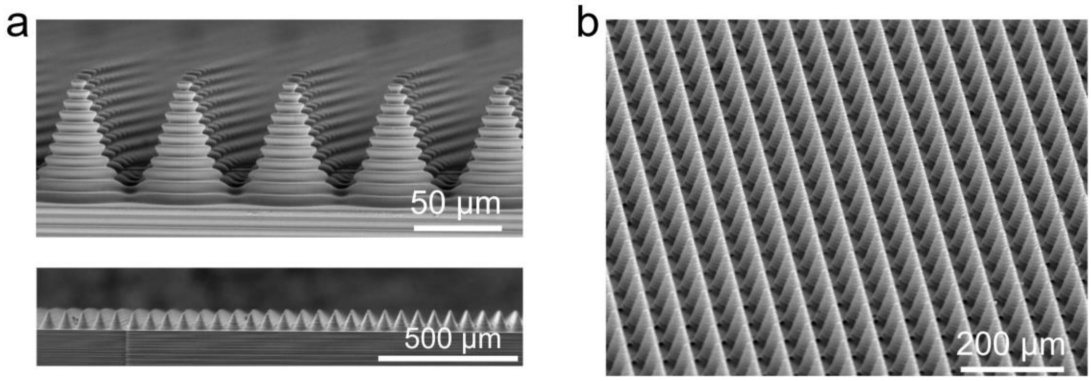

Supplementary Figure 1. SEM images of the template made by 3D printing. a, Cross-sectional view. b, Forty-five-degree tilt view.

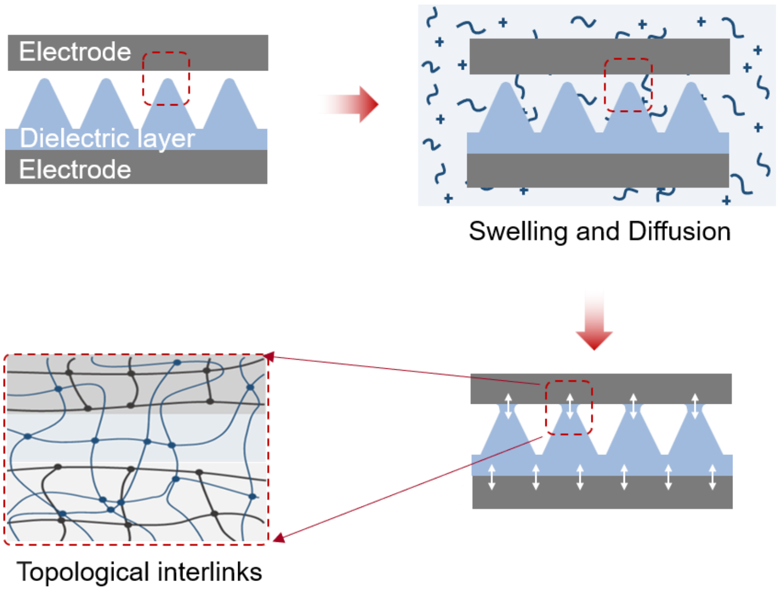

Supplementary Figure 2. **Schematic diagram illustrating the preparation process of the bonded interface.**

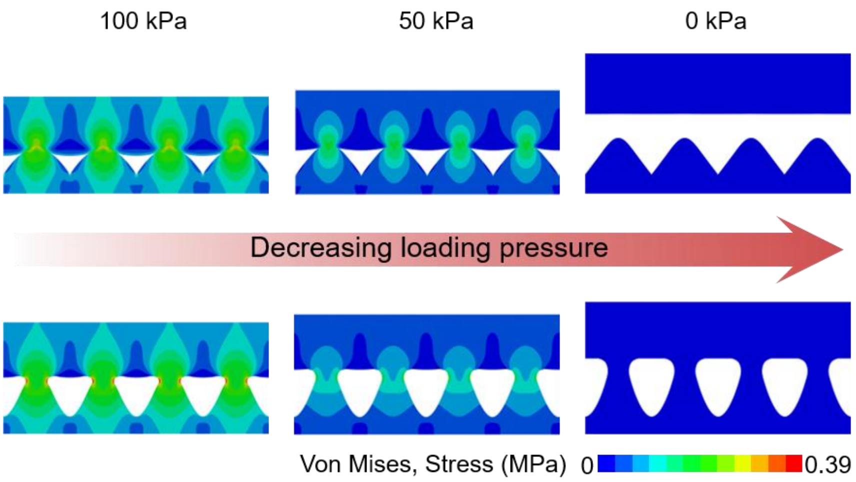

Supplementary Figure 3. Finite element simulations of both non-bonded and bonded pressure sensors under the unloading process.

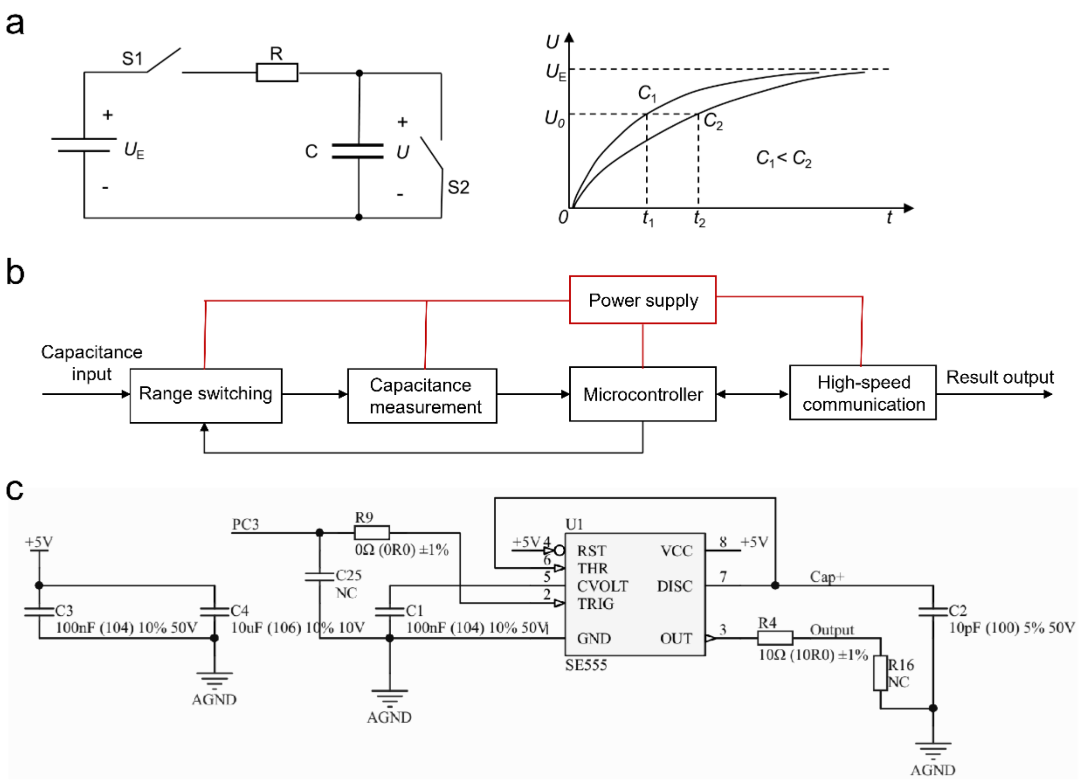

Supplementary Figure 4. Schematic design diagram of the digital circuit board. a, Schematic diagram of capacitor charging and discharging circuit and the correspondence between capacitance value and charging time. b, The capacitance measurement circuit that is mainly composed of five modules: power supply, range switching, capacitance measurement, microcontroller, and high-speed communication. c, Schematic diagram of capacitor charging and discharging circuit and the correspondence between capacitance value and charging time.

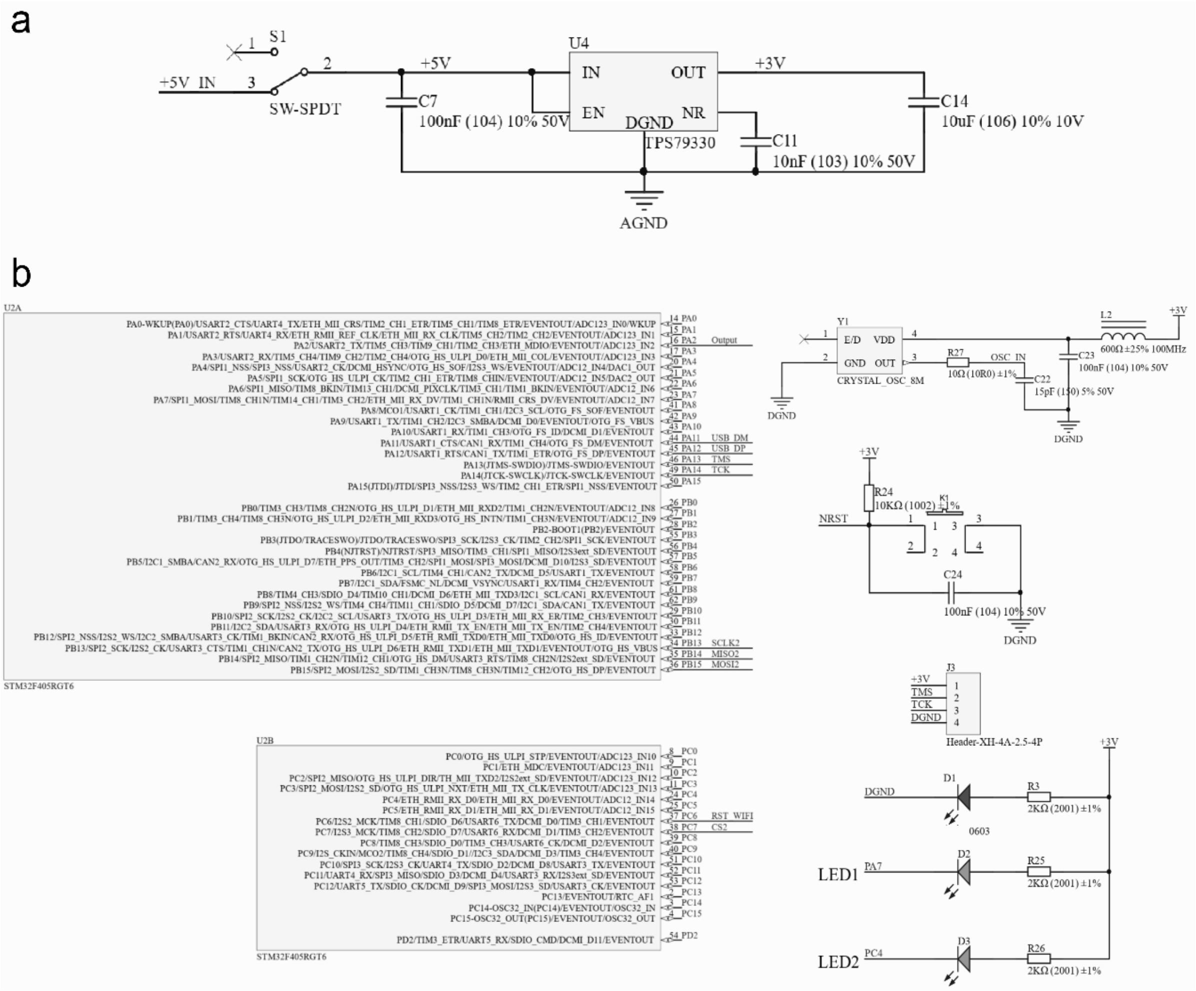

Supplementary Figure 5. Diagram of power supply module and microcontroller module of the digital circuit board. a, Schematic design diagram of power supply of the digital circuit board. b, Schematic design diagram of the microcontroller module of the digital circuit board.

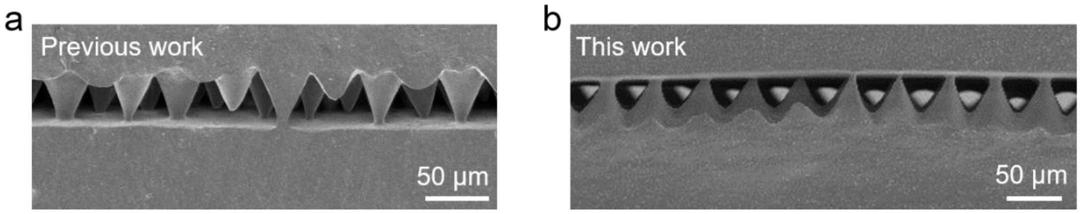

Supplementary Figure 6. SEM images of sensor structures with biomimetic structures and 3D printed structures. a, SEM image of cross-section of the sensor using biomimetic structures, showing a 70%~80% bonding ratio. b, SEM image of cross-section of the sensor in this work with a 100% bonding ratio.

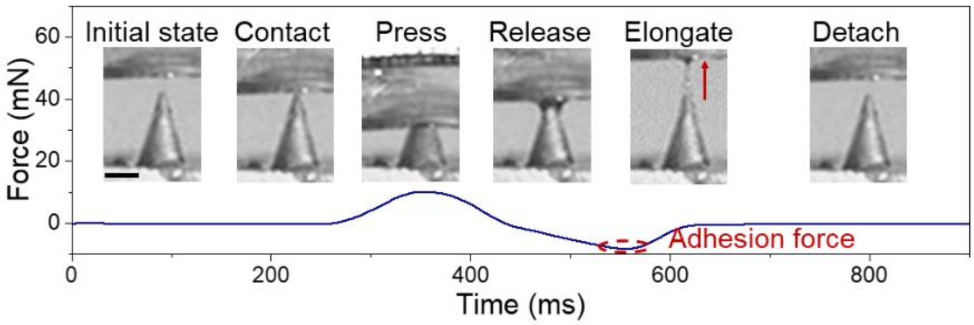

Supplementary Figure 7. Adhesion force between a PDMS cone and a PDMS thick layer during contact, pressing, and releasing. The scale bar is 500 μm.

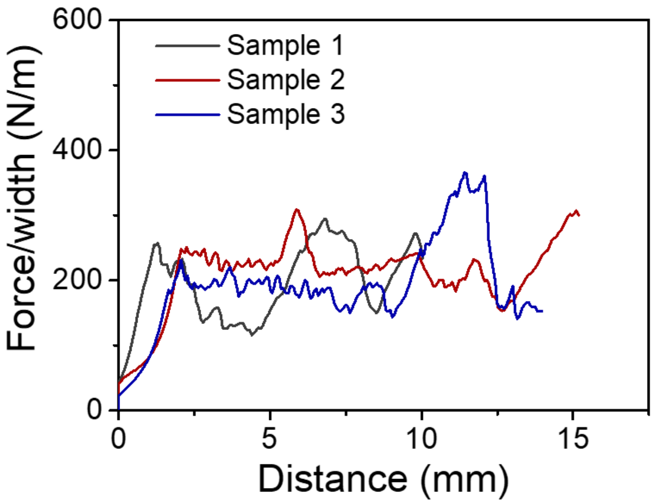

Supplementary Figure 8. Peel forces as a function of displacement for the three samples with bonded microstructured interface by 180° peeling.

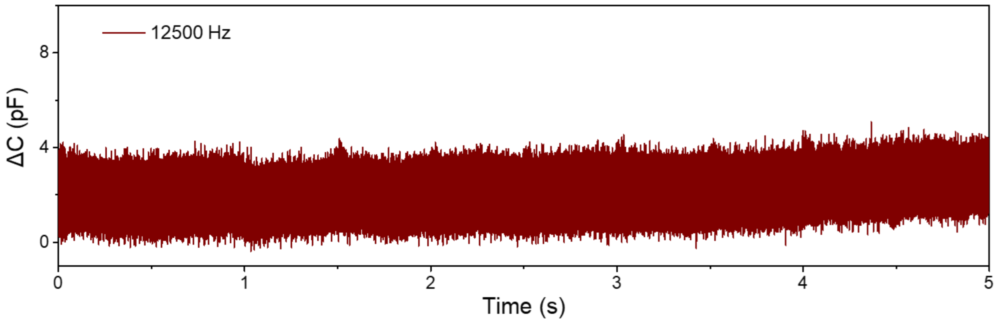

Supplementary Figure 9. Capacitive response of the sensor to 60,000 cycles of vibrations at a frequency of 12500 Hz.

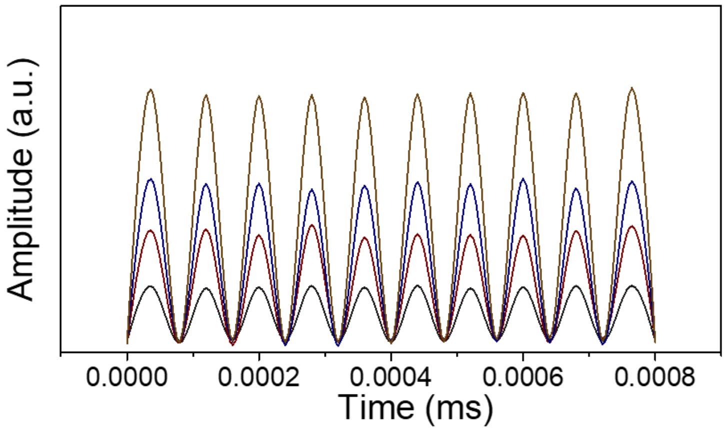

Supplementary Figure 10. Capacitive response of the pressure sensor to mechanical vibrations of different amplitudes at a fixed frequency of 12500 Hz.

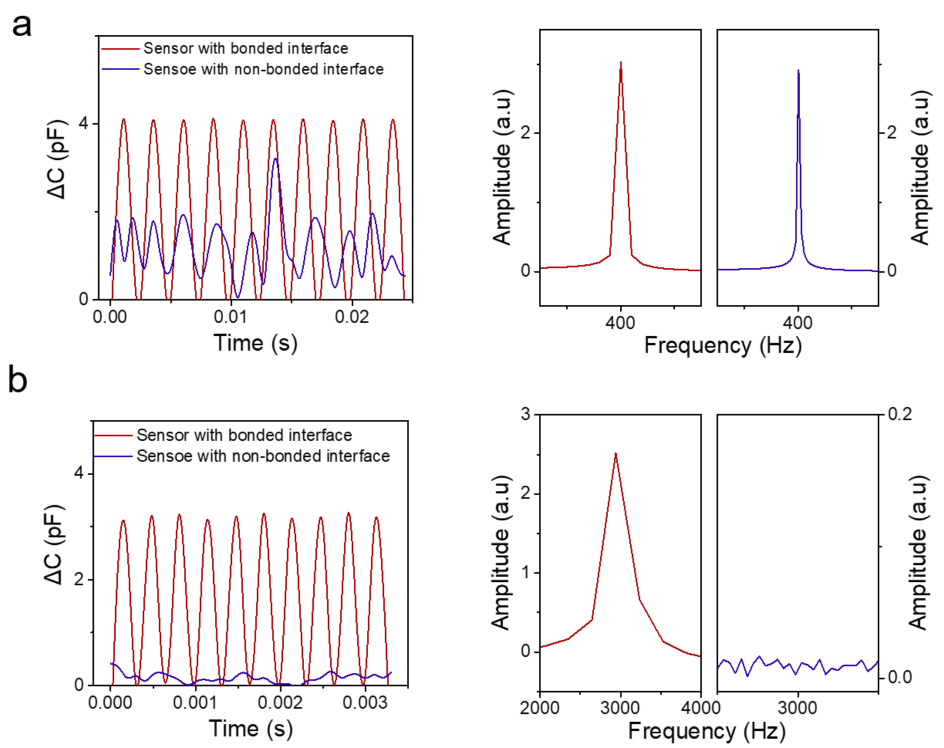

Supplementary Figure 11. Responses in both time and frequency domains of the sensors with and without interfacial bonding under vibrations of different frequencies. a, 400 Hz. b, 3000 Hz.

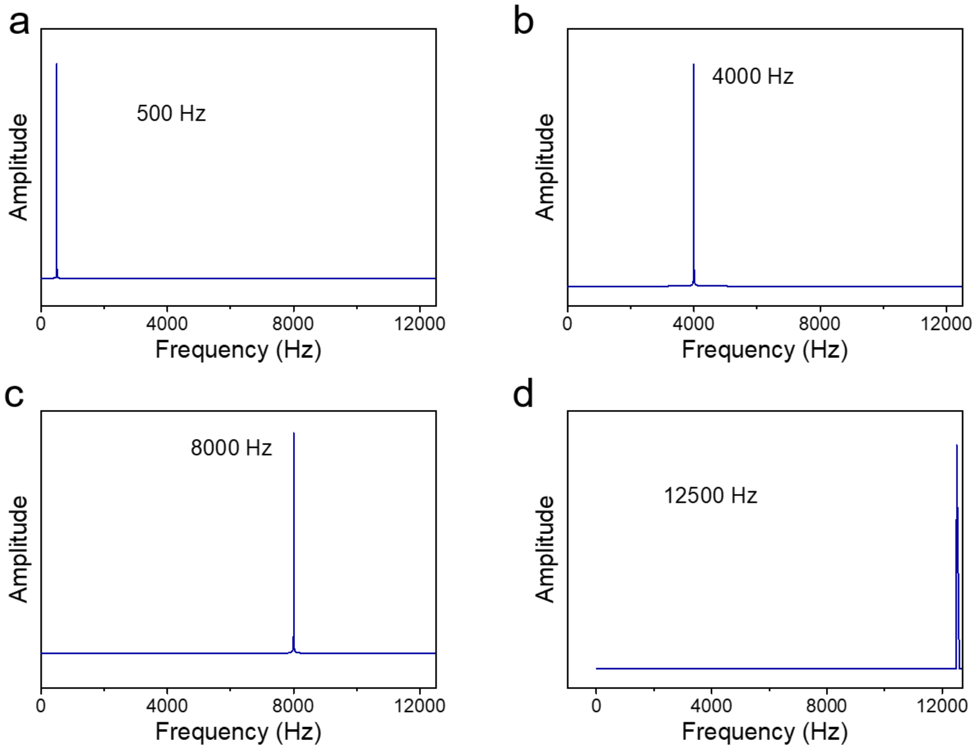

Supplementary Figure 12. Frequency spectra of the signals under a static pressure (100 kPa) and superposed vibrations of different frequencies. a, 500 Hz. b, 4000 Hz. c, 8000 Hz. d, 12000 Hz.

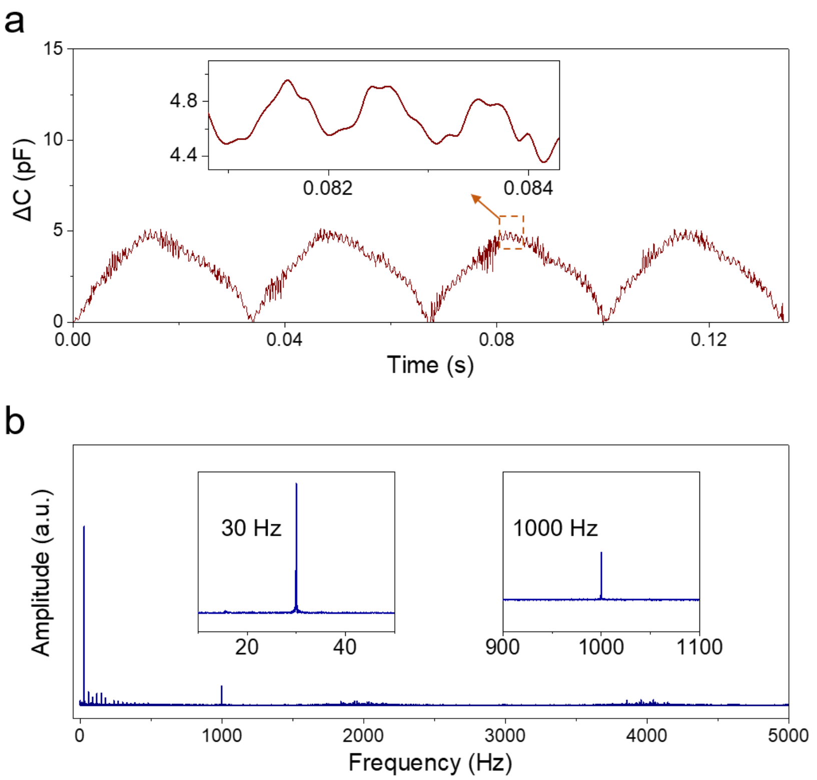

Supplementary Figure 13. Responses of our sensor to superposed vibrations. a, Capacitance response to superposed vibrations of 30 Hz and 1000 Hz. b, Corresponding frequency spectra of the signal, showing two peaks at 30 Hz and 1000 Hz.

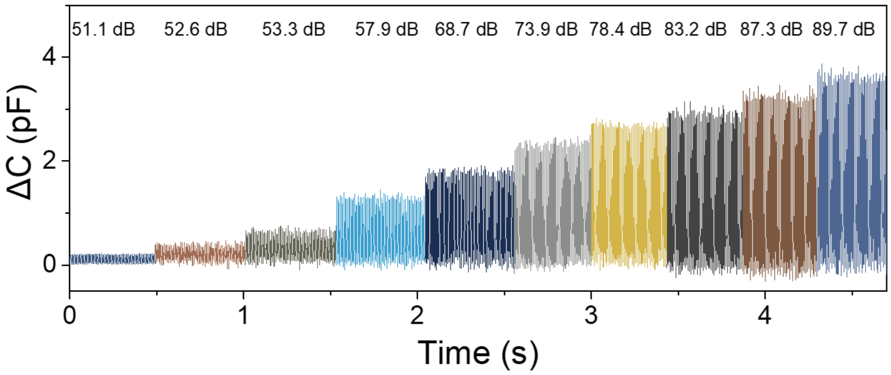

Supplementary Figure 14. Capacitance responses of the sensor to sounds of different sound pressure levels.

Supplementary Text 1. The design of digital circuit board. The capacitance measurement circuit is mainly composed of five modules: power supply, range switching, capacitance measurement, microcontroller, and high-speed communication (Supplementary Fig. 4b). The power module is used to provide a stable +3V DC power supply to other modules and consists mainly of a power switch and a low noise TPS79330 linear regulator. The capacitance measurement module is the heart of the circuit. As shown in Supplementary Fig. 4a, the capacitance value is measured via the charging time of the sensor under a specific DC voltage and resistance condition. A complete measurement cycle consists of capacitor charging and discharging. During the charging phase, switch S2 is open, and S1 is closed, controlled by a precision timer (SE555). The DC power supply U_E charges the measured capacitor C through the resistor R. The capacitance value is calculated by measuring the time that the capacitor takes to charge to a certain voltage U_0. During charging, the voltage across the measured capacitor rises exponentially with time. At a constant power supply voltage U_E and charging resistance R, the charging time depends solely on the measured capacitor. The larger the capacitor, the longer the charging time. Therefore, based on the measured charging time, the capacitance value can be calculated.

The capacitance measurement module uses a precision timer (SE555) to generate a pulse (Supplementary Fig. 4c). The duration of the high-level pulse generated by the module varies according to the capacitance being measured. When the SE555 receives a low-level control signal at the trigger input, it initiates the production of the high-level pulse. The duration of this high-level pulse, denoted as t_h, depends on the time constant of the R_C network.

$$t_h = 1.1R_A C = 1.1R_A(C_0 + C_e) \quad (1)$$

The charging and discharging resistance of the R_C network, denoted R_A, is finely tuned by the range switching module. This module provides four selectable levels of high precision resistors: 330, 100, 33, and 10 kΩ. In the R_C network, C is the total capacitance, consisting of the intrinsic capacitance C_0 of the circuit and the external capacitance C_e to be measured. Consequently, with fixed and known values of R_A and C_0, the measured capacitance C_e can be calculated by measuring the duration of the high-level pulse using

equation (1).

The microcontroller module controls the operation of the entire circuit and sends the measurement results via the communication module (Supplementary Fig. 5b). It consists of an STM32F405RGT6 microcontroller and peripheral circuits such as a quartz oscillator, reset, SWD download and LED display. When it is necessary to measure the capacitance, the MCU first receives the command from the external host computer to control the on-off of the range switching module relay to select the appropriate measurement range, then sends a low-level control signal to the capacitance measurement module, and at the same time starts the counter inside the MCU to start time the high-level pulse output by SE555, wait for the end of the high-level pulse to get its duration t_h, and calculate the measured capacitance value by formula (1) to complete a measurement.

The charging time for the capacitor to charge to a certain voltage U_0 is determined by U_E, R and C. According to the actual test results, the charging time exceeding 20 μs can avoid additional errors caused by too fast charging rates. For the charging time measurement accuracy, $C_{sensor} + C_{wires} + C_{circuit} \approx 200$ pF, and the minimum capacitance change that needs to be resolved is 0.05 pF. Therefore, the required resolution is at least 0.05/200×100% = 0.025%. The frequency of the precision timer is 1/168 μs. When the charging time is 25 μs, the count is 4200 times (25×168). The resolution is 1/4200×100% = 0.02%. In terms of capacitor discharge time, in order to avoid interference with the next capacitor measurement and cause cumulative errors, it is necessary to ensure that the voltage across the capacitor is discharged to zero as possible. Therefore, the discharging time is controlled above 10 μs. Therefore, considering all the above factors, the capacitance measurement period cannot be less than 25 + 10 = 35 μs, otherwise it will bring significant errors to the measurement. Taking into account the margin for other limiting factors such as data processing and switching time (5 μs), the measurement time is about 40 μs, resulting in a measurement frequency of 25 kHz.

Supplementary Text 2. Finite element simulation: Finite element analysis is performed using the commercial software package ABAQUS 2021. The finite element structure comprises a compression electrode layer and a microstructure dielectric layer, with materials designated as the electrode layer material (PDMS mixed with 7 wt.% CNTs) and the dielectric layer material (PDMS mixed with 2 wt.% CNTs). The electrode layer material is characterized as an approximately incompressible viscoelastic substance (Poisson's ratio = 0.46), featuring Young's modulus of 1 MPa, structural damping set at 0.2, and a density of 1 $g \cdot cm^{-3}$. Parameters for the dielectric layer material include properties of an approximately incompressible viscoelastic material (Poisson's ratio = 0.48), Young's modulus of 0.8 MPa, structural damping at 0.25, and a density of 0.985 $g \cdot cm^{-3}$. A dynamic explicit analysis step is defined, applying a constant speed loading to compress the electrode layer downward. The downward contact process is defined as a two-dimensional planar model problem, assuming all normal contacts are rigid without penetration. A frictional contact with a coefficient of friction set to 0.3 is introduced using a penalty function. The overall energy terms during the downward loading process include strain energy as the total energy term, and energy dissipation terms encompass frictional dissipation energy at the contact interface, viscoelastic dissipation energy, and viscous dissipation energy. Models of structural arrays with different bonding rates are established, varying contact surface length and the ratio of contact height to bottom length. The relevant models are then analyzed for energy terms and energy dissipation during the compression process. Additionally, a symmetrical array model with different ratios of contact surface length to bottom length is established to calculate the capacitance change during the compression process.

Detailed Modeling Process: We create structural array models with bonding rates ranging from 0% to 100%. These models feature adhesive contact surfaces with diverse diameter ratios (A_0/D) at 0.2, 0.4, 0.6, 0.8, and 1, as well as contact height ratios (H/D) at 0.5, 1, 1.5, and 2. For each bonding rate and combination of contact dimensions, the models underwent compression up to 100 kPa. Throughout this process, we meticulously calculate pertinent energy terms and energy dissipation items, offering a thorough analysis of the models' behavior. Furthermore, axisymmetric array models are

established with H/D=1, while A_0/D varied at 0.2, 0.4, 0.6, 0.8, and 1. These models underwent compression up to 400 kPa. Our focus is on scrutinizing changes in contact surface length (A_0) and height (H) during compression. This detailed examination enables us to quantify variations in structural capacitance under distinct compression scenarios. By systematically exploring the impact of bonding rates and diverse contact dimensions, this comprehensive modeling process provides valuable insights into the structural response and capacitance changes under specified pressure conditions.

Sensitivity calculation model:

In accordance with experimental data on the relative permittivity of the dielectric layer material during the compression process, as determined by Zhang,[1] a pressure (P) versus relative permittivity relationship curve was derived using exponential fitting:

$$\varepsilon = 98.11e^{0.0003532P} - 76.89e^{-0.02481P} \tag{2}$$

As depicted in Supplementary Fig. 15, the fitting results closely align with the experimental data. This indicates that the fitted curve can be effectively employed for the subsequent calculation of the relative permittivity of the dielectric layer.

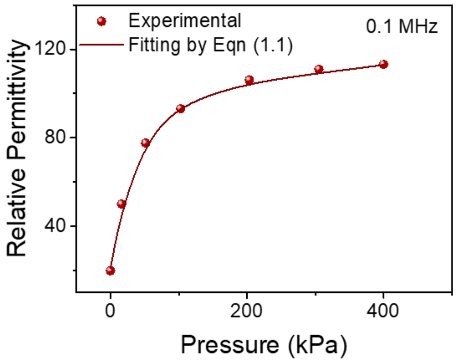

Supplementary Figure 15. Experimental data and fitting results for the relative permittivity of the dielectric layer material with respect to pressure.

In different axisymmetric models with varying lengths of bonded contact surfaces, the total capacitance C of the overall micro-cone structure comprises the capacitance of the dielectric layer C_d and the air capacitance C_{Air}, as depicted in Supplementary Fig. 16. Along the height direction, the micro-cone structure is divided into two segments: one is the cylindrical dielectric layer with a radius R, having a capacitance dC_d, and the other is the annular air layer with a capacitance dC_{Air}.

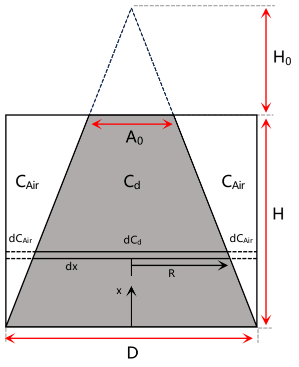

Supplementary Figure 16. **Calculation of geometric dimensions and capacitance for bonded micro-cone structure.**

Derived from geometric relationships:

$$\begin{cases} R = \dfrac{(H + H_0 - x)D}{2(H + H_0)} \\ H_0 = \dfrac{A_0 H}{D - A_0} \end{cases} \tag{3}$$

Then, the corresponding capacitances of the differential dielectric layer and air layer are:

$$\begin{cases} dC_d = \dfrac{\varepsilon \pi R^2}{dx} \\ dC_{Air} = \dfrac{\varepsilon_{Air} \pi (D^2 - 4R^2)}{4dx} \end{cases} \tag{4}$$

The two differential capacitances are in parallel, then the micro-layer capacitance of the overall structure is:

$$C = dC_d + dC_{Air} \tag{5}$$

The overall capacitance of the microstructure is equivalent to the series connection of each differential capacitance. Therefore, the total capacitance of the microstructure is the sum of the differential capacitances along the height direction:

$$C = \frac{1}{\sum {}^{1}/_{dC}} \tag{6}$$

Selecting the scenario with the maximum air capacitance, i.e., the model where the contact surface length to the bottom surface length ratio is A_0/D=0.2, the air capacitance and the initial capacitance of the overall structure are calculated. Since the relative permittivity (ε) of the dielectric layer under no load is 21.22, the calculation results indicate that the proportion of air capacitance is approximately 3.45%. Moreover, this proportion gradually decreases as the structure is compressed. Therefore, in the

subsequent calculation of the overall microstructure capacitance and sensitivity, we neglect the air capacitance and approximately equate the dielectric layer capacitance (C_d) to the overall structure capacitance (C).

During the loading process of the overall structure, the compressed height of the dielectric layer's top contracts, the bonded contact length gradually increases, and the overall capacitance progressively rises. Simultaneously, the overall pressure of the structure gradually increases, as shown in Supplementary Fig. 17. For cylindrical dielectric layers at different positions, pressures and relative permittivities are calculated using the form of equal forces on each cross-section:

$$\begin{cases} P_x = \dfrac{PD^2}{4R^2} \\ \varepsilon = 98.11e^{0.0003532P_x} - 76.89e^{-0.02481P_x} \end{cases} \tag{7}$$

Combining equation (3), we obtain expressions for the relative permittivity of cylindrical dielectric layers at different positions along the height direction.

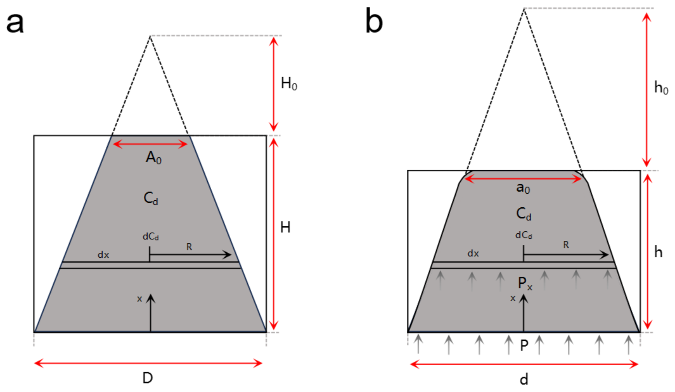

Supplementary Figure 17. Geometric dimensions and capacitance calculation before and after the compressed loading process of the bonded micro-cone structure. a, Bonded structure unloaded. b, Bonded loading process with pressure applied at the bottom as *P*.

Utilizing finite element simulations with varied contact length ratios, we derive the changes in contact length (a_0), height (h), and bottom pressure (P) of the bonded structure during loading up to 400 kPa. By integrating the aforementioned equations (3) - (7) and performing differential summation, we calculate the capacitance of the microstructure both before loading (C_0) and during loading (C). Subsequent data analysis enables the determination of the sensitivity ($\Delta C/C_0$) of the bonded microstructure concerning variations in loading pressure under different length ratios.

References

1. Zhang, Y., et al. Highly stable flexible pressure sensors with a quasi-homogeneous composition and interlinked interfaces. *Nat. Commun.* **13**, 1317 (2022).
